# Online Search Trends on Child Abuse During the COVID-19 Pandemic Using the Most Widely Used Search Engine: Retrospective Observational Study

**DOI:** 10.2196/93215

**Published:** 2026-06-23

**Authors:** Naoko Takada, Kosuke Ishizuka, Taiju Miyagami, Mizue Saita, Akihiko Kusakabe, Mitsuyasu Ohta, Toshio Naito

**Affiliations:** 1 Department of General Medicine School of Medicine Yokohama City University Yokohama Japan; 2 Department of General Medicine Faculty of Medicine Juntendo University Tokyo Japan

**Keywords:** abuse, child abuse, COVID-19 pandemic, infodemiology, online search trend

## Abstract

**Background:**

Child abuse is an important issue that threatens child development. The COVID-19 pandemic had psychological, social, and economic effects, and concerns were raised about a possible increase in child maltreatment.

**Objective:**

This study aimed to describe online search trends on child abuse in Japan during the COVID-19 pandemic.

**Methods:**

We conducted a retrospective observational study on the online search volume of terms related to abuse, such as “abuse,” “psychological abuse,” “physical abuse,” “neglect,” “sexual abuse,” “I might become abusive,” “I am scared I might become abusive,” and “I cannot stop abusing,” in Yahoo! Japan. Search volumes were compared with trends in the number of reported cases of COVID-19.

**Results:**

The number of searches for “neglect” increased during periods of social change caused by the COVID-19 pandemic and when waves of the pandemic coincided with the summer vacation period. Searches for terms suggesting abuse, such as “I might become abusive” and “I am scared I might become abusive,” increased in 2020 followed by a decrease from 2021 onward. Most of the searches were conducted by women aged 20 to 49 years.

**Conclusions:**

Online searches related to “neglect” increased during periods of major social change during the COVID-19 pandemic. Searches suggestive of concern about perpetrating abuse were conducted predominantly by women aged 20 to 49 years. Online search data may serve as a useful tool for identifying trends in child abuse–related search interest.

## Introduction

The COVID-19 pandemic started with an outbreak of pneumonia of unknown cause in Wuhan, China, in December 2019, eventually reaching Japan. Considerable knowledge has been accumulated on the symptoms of COVID-19, and diagnostic, therapeutic, and preventive methods have been established. However, the COVID-19 pandemic has not only had a devastating negative impact on health but has also had negative psychological, social, and economic effects [1,2], with an increase in the incidence of mental illness, suicide, and abuse of children and older adults [3-7]. Child abuse is a social problem that requires national countermeasures because of its serious adverse effects on children’s mental and physical health and personality development [8].

In Japan, according to Article 2 of the Child Abuse Prevention and Treatment Act, child abuse encompasses not only physical and sexual abuse but also neglect and psychological abuse [9]. According to the Ministry of Health, Labor, and Welfare and the Children and Families Agency, the number of consultations and responses to cases of child abuse has been increasing every year and reached a record high of 225,509 cases in 2023 [9]. The number of cases of abuse may have been underreported during the COVID-19 pandemic owing to school closures and voluntary lockdowns [4,10].

A previous study of online search trends during the COVID-19 pandemic compared the number of searches for “long COVID” with the number of new COVID-19 cases and found that online search trends were associated with real-world events [11]. The online search trends for changes over time in child maltreatment during the COVID-19 pandemic are unclear and, to our knowledge, have not been studied previously. Some studies suggest an increase in child abuse during the pandemic [4,10], whereas other studies suggest a decrease [12]; therefore, the actual situation is unclear.

The purpose of this study was to investigate the trend in online searches on child abuse in Japan and compare it with trends in the number of cases of COVID-19 reported.

## Methods

### Data Sources

To examine the patterns of online searches on abuse during the COVID-19 pandemic, we obtained data on online searches extracted from Yahoo! Japan, one of the most widely used search engines in Japan. Yahoo! Japan is the most accessed website in the country. According to Japanese data, the total number of accesses was 1.18 billion in November 2021.

Data on the monthly number of abuse-related online searches were available for the period from December 2019 to November 2023. Annual rankings of abuse-related search terms were available up to the top 500 terms for each year from 2019 to 2023.

Search volume data were obtained from Yahoo! Japan Corporation via DS.INSIGHT with legitimate access (last accessed on May 29, 2025). Yahoo! Japan DS.INSIGHT can be accessed by people working at educational and research institutions by obtaining a log-in ID. It is a tool that enables analysis of all Yahoo! Japan big behavioral data, such as keywords, period, gender, age, and prefecture and the transitions and trends in specific searches over time. The user manual is available [13]. The number of internet users in Japan was calculated based on the Communications Usage Trend Survey by the Ministry of Internal Affairs and Communications.

The number of new cases of COVID-19 in Japan was obtained from open access COVID-19 notification data on the Ministry of Health, Labor, and Welfare website. Comprehensive case count data were available only for the period from January 2020 to May 2023.

To compare with the monthly data on online searches related to abuse, the number of new COVID-19 cases was also aggregated on a monthly basis. Monthly search trend data were available for the period from December 2019 to November 2023, and visual comparisons of the monthly number of searches with monthly COVID-19 case counts were conducted from January 2020 to May 2023, the period for which both types of data were available.

### Search Queries Used in the Analysis

To investigate the trends in the number of online searches on abuse, we conducted an observational study using the volume of search terms obtained from Yahoo! Japan. We used the search terms “abuse,” “psychological abuse,” “physical abuse,” “neglect,” and “sexual abuse” to evaluate online interest in abuse based on monthly data available from December 2019 to November 2023, with the last 4 terms corresponding to the 4 categories of child abuse used on the official website of the Administration for Children and Families under the Law Concerning the Prevention of Child Abuse. In addition, to clarify the trends in the parties conducting searches related to abuse, we analyzed the top 500 search terms including “abuse” in 2020, the year in which the COVID-19 pandemic began, and for each query, we observed the trends for each year from 2019 to 2023 for which top 500 data were available.

Queries related to anticipated or ongoing perpetration of abuse were extracted independently by 2 evaluators (NT and KI), and a third evaluator (MO) also provided an assessment to minimize the effect of observer bias. The search terms “I might become abusive” and “I am scared I might become abusive,” which suggest anticipated perpetration of abuse, and “I cannot stop abusing,” which suggests perpetrating abuse at the time of the search, were identified. We also identified the query “I have abused”; however, this query was excluded from the main trend analysis because its Japanese equivalent is semantically and temporally ambiguous and may indicate either an abusive act committed in the past with continuity to the present or regret over an abusive act committed in the past without continuity to the present and the annual search volume did not show a clear temporal pattern.

The designation as “Out of ranking,” which is shown in the Results section, indicates that a query was not ranked in the top 500 searches for that period and that the search volume was lower than that of the 500th-ranked query.

### Statistical Analysis

Details regarding Yahoo! Japan’s internal statistical processing were not disclosed owing to concerns that individual data might be identified. Internet users were stratified by age, gender, and year on the day of the search. Categorical variables were summarized as frequencies and percentages. Descriptive statistics were compiled using Microsoft Excel 2019.

### Ethical Considerations

This study adhered to Chapter 1, Part 3, Section 1, under category (C), item (ⅲ) of the Ethical Guidelines for Medical and Biological Research Involving Human Subjects of the Ministry of Health, Labour and Welfare of Japan [14]. In accordance with this guideline, since this study used previously anonymized and de-identified data, an ethical review was waived, and patient informed consent was not required.

## Results

Between 2019 and 2023, between 50.3% (49,883,262/99,252,024) and 51% (48,512,132/95,187,232) of all internet users in Japan were men, and the user population was evenly distributed among different age groups (Table 1). The number of online searches related to abuse decreased between 2019 and 2023, with fluctuations from year to year. The gender distribution showed that online searches related to abuse were more commonly conducted by women (40,600/59,200, 68.6%-48,100/66,200, 72.7%) than by men (18,100/66,200, 27.3%-18,600/59,200, 31.4%), and the age distribution showed that the 30- to 49-year age group conducted more online searches related to abuse than other age groups, with 18.2% (10,800/59,200) to 28.1% (32,200/114,600) of searchers aged 30 to 39 years and 22.2% (16,200/73,100) to 24.3% (27,800/114,600) aged 40 to 49 years (Table 1).

**Table 1:** Number of online searches using the Yahoo! Japan search engine that included the term “abuse” by gender, age, and year (2019 to 2023).

			2019				2020				2021			2022				2023			
			Internet users^a^, n (%)		Search term “abuse”^b^, n (%)		Internet users^c^, n (%)		Search term “abuse”^d^, n (%)		Internet users^e^, n (%)	Search term “abuse”^f^, n (%)		Internet users^g^, n (%)			Search term “abuse”^h^, n (%)	Internet users^i^, n (%)			Search term “abuse” ^j^, n (%)
**Gender**																					
	Man	51,488,428 (50.4)		33,900 (29.6)		48,512,132 (51)		18,100 (27.3)		49,333,623 (50.6)			20,700 (28.3)		49,883,262 (50.3)	19,600 (28.9)			50,267,774 (50.6)	18,600 (31.4)	
	Woman	50,755,843 (49.6)		80,700 (70.4)		46,675,100 (49)		48,100 (72.7)		48,155,775 (49.4)			52,400 (71.7)		49,368,762 (49.7)	48,300 (71.1)			49,099,369 (49.4)	40,600 (68.6)	
**Age group (y)**																					
	<20	13,497,868 (13.2)		13,700 (12)		13,304,833 (14)		9800 (14.8)		13,686,376 (14)			12,300 (16.8)		13,467,288 (13.6)	8900 (13.1)			13,376,729 (13.5)	6700 (11.3)	
	20-29	12,281,904 (12)		16,700 (14.6)		12,291,295 (12.9)		9700 (14.7)		12,293,873 (12.6)			9600 (13.1)		12,406,690 (12.5)	7500 (11)			12,228,406 (12.3)	5400 (9.1)	
	30-39	13,715,342 (13.4)		32,200 (28.1)		13,721,738 (14.4)		18,600 (28.1)		13,364,445 (13.7)			16,800 (23)		13,293,144 (13.4)	14,700 (21.6)			13,037,003 (13.1)	10,800 (18.2)	
	40-49	17,603,599 (17.2)		27,800 (24.3)		17,368,194 (18.2)		15,100 (22.8)		17,150,128 (17.6)			16,200 (22.2)		16,718,688 (16.8)	15,600 (23)			16,143,714 (16.2)	13,700 (23.1)	
	50-59	15,496,656 (15.2)		12,800 (11.2)		15,320,558 (16.1)		7400 (11.2)		15,934,650 (16.3)			10,300 (14.1)		16,398,504 (16.5)	11,300 (16.6)			16,873,071 (17)	11,600 (19.6)	
	60-69	13,792,110 (13.5)		6300 (5.5)		12,116,356 (12.7)		3600 (5.4)		12,497,583 (12.8)			5100 (7)		12,735,846 (12.8)	6300 (9.3)			12,920,129 (13)	7000 (11.8)	
	≥70	15,856,792 (15.5)		5100 (4.5)		11,064,258 (11.6)		2000 (3)		12,562,343 (12.9)			2800 (3.8)		14,231,864 (14.3)	3600 (5.3)			14,788,091 (14.9)	4000 (6.8)	

^a^n=102,244,271.

^b^n=114,600.

^c^n=95,187,232.

^d^n=66,200.

^e^n=97,489,398.

^f^n=73,100.

^g^n=99,252,024.

^h^n=67,900.

^i^n=99,347,143.

^j^n=59,200.

**Figure 1:**
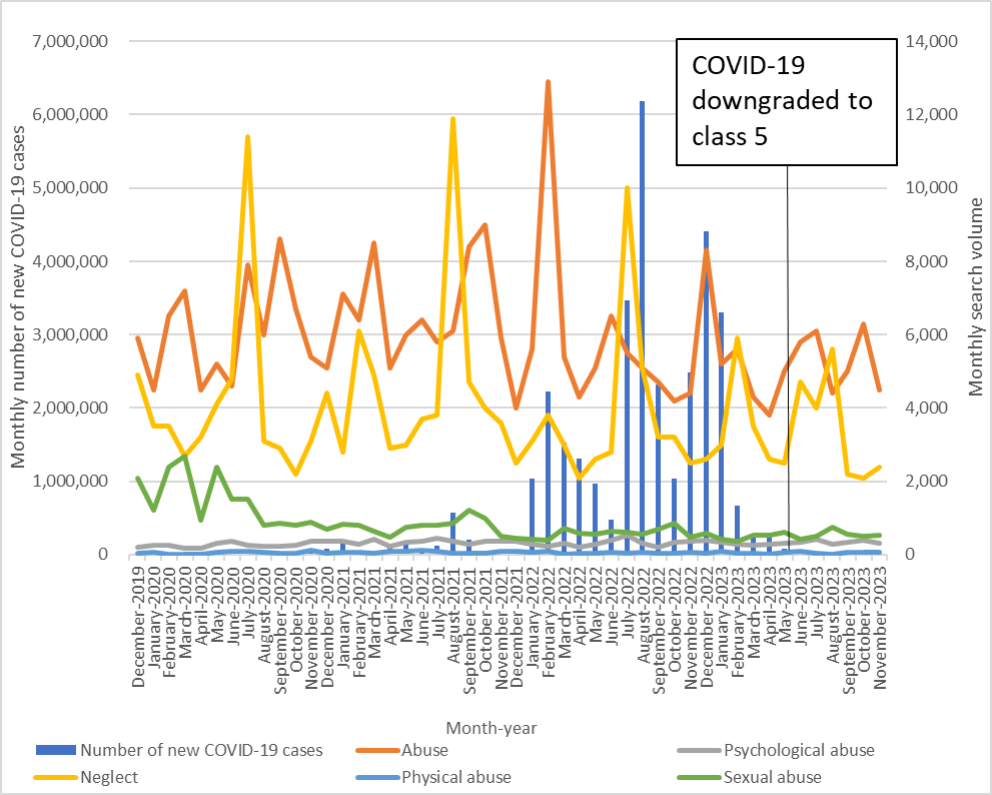
Monthly number of new COVID-19 cases (January 2020 to May 2023) and monthly search volume using the terms “abuse,” “psychological abuse,” “neglect,” “physical abuse,” and “sexual abuse” (December 2019 to November 2023). On May 8, 2023, the Japanese government reclassified COVID-19 as a class 5 infectious disease according to the Infectious Diseases Control Law, placing it on the same level as seasonal influenza and syphilis.

On visual comparison of monthly trends, no clear relationship was apparent between the number of new COVID-19 cases and the search volume for “abuse,” “psychological abuse,” “physical abuse,” “neglect,” and “sexual abuse”; however, the number of searches for “neglect” increased during the early phase of the COVID-19 pandemic (from March 2020 to August 2020), the fifth wave (June to November 2021), and the seventh wave (June to October 2022). During the study period, the number of new COVID-19 cases increased from year to year, but the number of searches for “neglect” did not (Figure 1).

Next, we extracted data on searches that suggested perpetration of abuse and found that the search volume for “I might become abusive” increased from 1300 in 2019 to 1500 in 2020 (a 15.4% increase) and then decreased to 1100 in 2021 (a 26.7% decrease from 2020). For “I am scared I might become abusive,” the exact 2019 search volume was unavailable because the term was reported as out of ranking (≤410 searches); therefore, a percentage change could not be calculated. However, the search volume was 460 in 2020, indicating that the number of searches for this term also increased from 2019 to 2020. It then decreased to 310 in 2021 (a 32.6% decrease from 2020). Women accounted for 94% and 96% of those searching for “I might become abusive” and “I am scared I might become abusive,” respectively, in 2020, and internet users aged 20 to 49 years accounted for 96% and 89% of those searching for “I might become abusive” and “I am scared I might become abusive,” respectively, in 2020 (Table 2). Of those searching for “I cannot stop abusing,” suggesting ongoing abuse, women accounted for 88% of the searchers by gender, and internet users aged 20 to 49 years accounted for 92% of searchers by age. The annual number of online searches for “I cannot stop abusing” decreased by 12.6% in 2020 compared with 2019 and by 6% in 2021 compared with 2020, indicating a decrease in the number of searches conducted during the COVID-19 pandemic.

**Table 2:** Number of searches related to committing abuse by gender, age, and year (2019 to 2023). Since Yahoo! Japan uses expanded statistics, this table lists only the total number of searches and the percentage.

Search term and year		Search volume (persons), n	Man (%)	Woman (%)	10s and below (%)	20s (%)	30s (%)	40s (%)	50s (%)	60s (%)	70s and older (%)
“**I might become abusive”**											
	2019	1300	5	95	1	24	60	10	1	1	3
	2020	1500	6	94	1	21	62	13	1	1	1
	2021	1100	4	96	4	20	60	13	1	1	1
	2022	880	6	94	2	18	62	15	1	1	1
	2023	810	6	94	2	15	63	17	1	1	1
“**I am scared I might become abusive”**											
	2019	Out of ranking: search volume (persons) of 410 or less	Out of ranking: search volume (persons) of 410 or less	Out of ranking: search volume (persons) of 410 or less	Out of ranking: search volume (persons) of 410 or less	Out of ranking: search volume (persons) of 410 or less	Out of ranking: search volume (persons) of 410 or less	Out of ranking: search volume (persons) of 410 or less	Out of ranking: search volume (persons) of 410 or less	Out of ranking: search volume (persons) of 410 or less	Out of ranking: search volume (persons) of 410 or less
	2020	460	4	96	7	20	54	15	2	2	0
	2021	310	6	94	0	19	59	19	3	0	0
	2022	Out of ranking: search volume (persons) of 290 or less	Out of ranking: search volume (persons) of 290 or less	Out of ranking: search volume (persons) of 290 or less	Out of ranking: search volume (persons) of 290 or less	Out of ranking: search volume (persons) of 290 or less	Out of ranking: search volume (persons) of 290 or less	Out of ranking: search volume (persons) of 290 or less	Out of ranking: search volume (persons) of 290 or less	Out of ranking: search volume (persons) of 290 or less	Out of ranking: search volume (persons) of 290 or less
	2023	Out of ranking: search volume (persons) of 280 or less	Out of ranking: search volume (persons) of 280 or less	Out of ranking: search volume (persons) of 280 or less	Out of ranking: search volume (persons) of 280 or less	Out of ranking: search volume (persons) of 280 or less	Out of ranking: search volume (persons) of 280 or less	Out of ranking: search volume (persons) of 280 or less	Out of ranking: search volume (persons) of 280 or less	Out of ranking: search volume (persons) of 280 or less	Out of ranking: search volume (persons) of 280 or less
“**I cannot stop abusing”**											
	2019	950	11	89	3	18	55	19	2	2	1
	2020	830	12	88	5	18	52	22	1	2	0
	2021	780	8	92	3	17	51	24	3	1	1
	2022	660	9	91	0	17	49	27	3	2	2
	2023	650	9	91	5	12	46	26	5	3	3

## Discussion

### Principal Findings

To contribute to the understanding of the epidemiology of child abuse in Japan, this study characterized the trends in online searches related to child abuse during the COVID-19 pandemic.

According to information on the Administration for Children and Families website, the number of consultations on child abuse at Child Guidance Centers in Japan has been increasing each year; however, this increase was not reflected in the number of online searches related to child abuse during the 5-year study period. Furthermore, the number of searches for “psychological abuse,” which accounted for 59.6% of all consultations on child abuse at Child Guidance Centers in 2022 (according to information on the Administration for Children and Families website), was small, whereas the number of searches for “neglect,” which accounted for only 16.2% of all consultations on child abuse at Child Guidance Centers in 2022, was the largest of the 4 child abuse categories considered, showing a discrepancy between the number of consultations related to specific types of child abuse and the volume of online searches for the corresponding type of abuse. Possible reasons for this discrepancy may be that online searches are influenced by news reports of prominent cases and that online searches include searches for cases that are not clearly abusive because the general perception of what constitutes abuse among internet users is broader than the legal definition of abuse. In this study, on visual comparison of monthly trends, no clear relationship was apparent between the number of new COVID-19 cases and the search volumes for the terms “abuse,” “psychological abuse,” “physical abuse,” “neglect,” and “sexual abuse.”

The observed increase in searches for “neglect” may be interpreted in the context of social changes during the COVID-19 pandemic. Previous studies have identified economic factors, mental and physical health problems of parents and children, and parenting stress as risk factors for neglect [15]. The COVID-19 pandemic led to increased parenting burden owing to parents’ economic difficulties, worsening mental health, and social isolation [16-19]. Previous studies on child abuse during the COVID-19 pandemic have reported mixed findings [20], and no consistent trend has been established for specific types of abuse. Although this has not been a conclusion consistently supported across the existing literature, some studies have suggested an increase in the incidence of neglect [21,22].

In Japan, during the period of voluntary curfew, worsening of physical and mental symptoms was especially noticeable among young adults aged 18 to 29 years; women; people with a history of treatment for mental illness; and people with socially disadvantaged backgrounds, including those with an annual income of less than ¥2 million (US $12,400; ¥1=US $0.0062 as of June 8, 2026) [23]. In another study of pregnant women and mothers less than a year post partum conducted during the COVID-19 pandemic [24], 38% reported “decreased income,” 77.2% reported “fear of COVID-19,” and 18.1% reported “experiencing criticism for taking my child to a public place.” Of these, “decreased income” and “experiencing criticism for taking my child to a public place” were associated with a higher risk of perinatal depression [24]. These prior findings suggest that some factors associated with neglect may have worsened during the COVID-19 pandemic, providing a possible context for interpreting the observed increase in neglect-related searches.

In Japan, COVID-19 began to have an impact on daily life in March 2020. All elementary, junior high, and high schools in Japan were temporarily closed during spring break starting on March 2, 2020, and on April 7, 2020, a state of emergency was declared, requiring voluntary restraint from going out and holding events and restricting the use of facilities, which changed the way of life. The state of emergency was lifted on May 25, 2020, and the restrictions were gradually lifted in June 2020, but the number of cases of COVID-19 began to increase, leading directly to the second wave. The number of searches for “neglect” gradually increased starting in March 2020 followed by a decrease starting in August 2020, when the second wave began to subside. During the fifth wave (June to November 2021) and the seventh wave (June to October 2022), the number of searches for “neglect” increased markedly and then decreased as the number of new COVID-19 cases declined. Both the fifth and seventh waves occurred during the summer vacation period in Japan. During school vacations and natural disasters, children are isolated from their communities and schools, and programs to prevent child abuse are canceled; therefore, abuse is less likely to be reported despite an increased incidence [25,26]. Nevertheless, the results of this study show that online interest in “neglect” increased even during such periods.

During the seventh wave, the number of new cases of COVID-19 was higher than that during the fifth wave, but the number of online searches using the term “neglect” was lower. Changes between the fifth and seventh waves included a shift from the Delta variant to the less virulent Omicron variant and the holding of traditional festivals in various parts of Japan for the first time in 3 years, which eased the mood of self-restraint and led to a recovery in socioeconomic activities. Furthermore, in September 2022, the government introduced a shortened isolation period for individuals with COVID-19. After the eighth wave, on visual comparison, the number of searches for “neglect” did not appear to increase to the same extent as that observed during the early phase of the COVID-19 pandemic and during the fifth and seventh waves.

With each successive lockdown in Japan, the tendency for exacerbation of physical and mental symptoms was less marked owing to adaptation to the environmental changes brought about by the COVID-19 pandemic [23]. This study demonstrates that, although the annual number of new COVID-19 cases increased from thousands in 2020 to hundreds of thousands in 2022, the number of searches for “neglect” did not show a corresponding increase. Although the number of searches for “neglect” was not proportional to the number of new cases of COVID-19, the number of searches for “neglect” increased during periods of changes in living environments due to the COVID-19 pandemic, such as during the period of state emergency declarations and measures to prevent the spread of the disease and summer vacation periods coinciding with the COVID-19 pandemic waves.

These results are consistent with those of previous studies [15-19] that identified economic factors, mental and physical health problems of parents and children, and parenting stress as risk factors for neglect, all of which were amplified during the COVID-19 pandemic.

As searches for “abuse,” “psychological abuse,” “physical abuse,” “neglect,” and “sexual abuse” include searches from searchers with diverse intentions, we investigated searches that may reflect concern about potential or ongoing perpetration of abuse taking advantage of the anonymity of online searches.

We focused on searches from 2020 to 2021, the period immediately after the onset of the COVID-19 pandemic, and found that the number of searches for “I might become abusive” and “I am scared I might become abusive,” which may reflect concern about potential perpetration of abuse, increased in 2020 and decreased in 2021 compared with 2019 and 2020, respectively. These searches were mainly conducted by women in their 20s to 40s, an age group likely to include mothers. In 2020, shortly after the onset of the COVID-19 pandemic, when lifestyle changes were most marked, the number of searches on the fear of perpetrating abuse increased the most among young adults, the age group most likely to include parents of young children.

According to the surveys conducted by the Japan Institute for Labor Policy and Training in 2012 and 2014, a total of 12% of mothers in Japan are worried that they may be abusing their children even during normal times, and 40.5% of those who worry about being abusive engage in excessive physical punishment or neglect [27]. These tendencies may have intensified in 2020. However, searches for “I cannot stop abusing,” which suggests ongoing abuse-related concern at the time of the search, were predominantly by women aged 20 to 49 years, an age group likely to include mothers, but the increase did not correspond to the COVID-19 restrictions or to waves of the COVID-19 pandemic. On the basis of these findings, we assume that, at the onset of the COVID-19 pandemic in 2020, which led to marked changes in lifestyle, the number of women aged 20 to 49 years who searched for terms suggesting concern about becoming perpetrators of abuse increased, whereas searches suggesting ongoing abuse did not show a corresponding increase. However, based on these results, the possibility of changes in the degree or type of abuse during the COVID-19 pandemic cannot be ruled out.

This study found that the internet was also a means of accessing information for women in their 20s to 40s, an age group that may include mothers, who were concerned about perpetrating abuse. According to the World Health Organization handbook on measuring and monitoring national prevalence of child maltreatment, the results of surveys of parents regarding abuse are influenced by denial of socially undesirable behavior, leading to underreporting and inconsistencies with surveys of children; therefore, anonymity in fact-finding surveys is important [28]. Because anonymity is ensured, monitoring online search trends may be a useful tool for understanding changes in online interest in or concern about abuse-related issues during a pandemic.

### Limitations

This study has several limitations. First, Yahoo! Japan was the sole data source, and online searches conducted using other search engines were not considered; therefore, the results may not be generalizable beyond the specific population from which the sample was drawn. Second, data obtained from online search engines may be subject to nonrepresentative sampling and methodological biases specific to search platforms. Third, no consideration was given to other factors that affect online interest in abuse, such as large media coverage, school closures, seasonal factors, and broader help-seeking behavior, because this was not possible. Fourth, the available data were limited in temporal scope, and comparisons across data sources were restricted to the period in which they overlapped. Fifth, annual rankings of abuse-related search terms were available only for the top 500 terms for each year, which limited the ability to follow some terms continuously across the 5-year study period. Furthermore, potentially relevant but less frequently searched terms may have been excluded.

### Conclusions

On visual comparison, the observed increase in searches for the term “neglect” during the COVID-19 pandemic may be interpreted in the context of social changes during this period. Although no direct causal relationship could be established, this pattern may reflect the influence of factors identified in previous studies, including psychosocial stress, economic hardship, and social isolation. Previous studies have shown that, during school vacations and natural disasters, children are more isolated from society, making reporting of abuse less likely, but online interest in neglect increased during school vacations and during periods coinciding with waves of the COVID-19 pandemic. In addition, searches by women in their 20s to 40s, an age group that may include mothers, suggesting concern about perpetrating abuse also showed an increase in 2020 at the onset of the COVID-19 pandemic. Monitoring online search data is useful for understanding patterns of online interest in abuse as such searches can be conducted anonymously.

## Data Availability

The raw dataset supporting the conclusions of this paper is available from the corresponding author upon request.
